# Highly expressed captured genes and cross-kingdom domains present in Helitrons create novel diversity in *Pleurotus ostreatus* and other fungi

**DOI:** 10.1186/1471-2164-15-1071

**Published:** 2014-12-05

**Authors:** Raúl Castanera, Gúmer Pérez, Leticia López, Rubén Sancho, Francisco Santoyo, Manuel Alfaro, Toni Gabaldón, Antonio G Pisabarro, José A Oguiza, Lucía Ramírez

**Affiliations:** Department of Agrarian Production, Genetics and Microbiology Research Group, Public University of Navarre, 31006 Pamplona, Navarre, Spain; Bioinformatics and Genomics Programme, Centre for Genomic Regulation (CRG), Barcelona, Spain; Universitat Pompeu Fabra (UPF), 08003 Barcelona, Spain; Institució Catalana de Recerca i Estudis Avançats (ICREA), Pg. Lluís Companys 23, 08010 Barcelona, Spain

**Keywords:** Helitron, Transposable element, Gene expression, Gene capture, *Pleurotus ostreatus*, Helicase, Basidiomycete, Genome structure

## Abstract

**Background:**

Helitrons are class-II eukaryotic transposons that transpose via a rolling circle mechanism. Due to their ability to capture and mobilize gene fragments, they play an important role in the evolution of their host genomes. We have used a bioinformatics approach for the identification of helitrons in two *Pleurotus ostreatus* genomes using *de novo* detection and homology-based searching. We have analyzed the presence of helitron-captured genes as well as the expansion of helitron-specific helicases in fungi and performed a phylogenetic analysis of their conserved domains with other representative eukaryotic species.

**Results:**

Our results show the presence of two helitron families in *P. ostreatus* that disrupt gene colinearity and cause a lack of synteny between their genomes. Both putative autonomous and non-autonomous helitrons were transcriptionally active, and some of them carried highly expressed captured genes of unknown origin and function. In addition, both families contained eukaryotic, bacterial and viral domains within the helitron’s boundaries. A phylogenetic reconstruction of RepHel helicases using the Helitron-like and PIF1-like helicase conserved domains revealed a polyphyletic origin for eukaryotic helitrons.

**Conclusion:**

*P. ostreatus* helitrons display features similar to other eukaryotic helitrons and do not tend to capture host genes or gene fragments. The occurrence of genes probably captured from other hosts inside the helitrons boundaries pose the hypothesis that an ancient horizontal transfer mechanism could have taken place. The viral domains found in some of these genes and the polyphyletic origin of RepHel helicases in the eukaryotic kingdom suggests that virus could have played a role in a putative lateral transfer of helitrons within the eukaryotic kingdom. The high similarity of some helitrons, along with the transcriptional activity of its RepHel helicases indicates that these elements are still active in the genome of *P. ostreatus*.

**Electronic supplementary material:**

The online version of this article (doi:10.1186/1471-2164-15-1071) contains supplementary material, which is available to authorized users.

## Background

Transposable elements (TEs) are involved in genome organization, chromosomal rearrangements and changes in gene structure and expression. TEs are classified into two classes based on their mode of transposition [1]. Class I includes elements that transpose via RNA intermediates. This class can be further divided based on the presence or absence of long terminal repeats (LTR elements and non-LTR elements). Class II encompasses elements that transpose directly from DNA to DNA, a reaction catalyzed by a transposase. Class II TEs usually harbor terminal inverted repeats (TIRs) and create target site duplications TSDs during transposition. The different TE classes encompass autonomous elements, which contain all of the proteins necessary for transposition, and non-autonomous elements, which are defective copies resulting from deletions, insertions or rearrangements that affect the internal sequence. Thus, the transposition of non-autonomous elements relies on proteins encoded by autonomous copies. Recently, a novel group of Class II DNA TEs called helitrons was detected in *Arabidopsis thaliana* and *Caenorhabditis elegans* by a repeat-based computational analysis [2]. Helitrons are rolling-circle transposons that have been found in plants, protozoans, fungi, cnidarians, insects, worms, fishes, frogs, reptiles and mammals [2–6]. These elements are characterized by their 5′TC and 3′CTRR conserved ends as well as a 16- to 20-nucleotide hairpin-forming sequence located approximately 12 nucleotides upstream of the 3′CTRR end [2]. Helitrons lack TIRs, do not generate TSDs upon insertion, and are thought to transpose through a replicative rolling circle (RC) mechanism [2, 7] similar to that of bacterial IS91 elements [8]. Nevertheless, footprints of helitron somatic excisions have been recently reported in the maize genome, indicating that they may exhibit both replicative and excision-mediated modes of transposition [9]. Putative autonomous helitrons contain genes encoding a RepHel protein with a rolling-circle replication initiator (Rep) and a helicase (Hel) domain. Both domains are thought to be essential for transposition. The Rep domain is most likely involved in endonucleolytic DNA breaks during the excision and religation of the transposed DNA [3]. The Hel domain encodes a 5′-3′ DNA helicase in the PIF1/RRM3 family that is highly conserved from yeasts to humans and contributes to the maintenance of genome stability [10]. When helitrons transpose, they are inserted into AT dinucleotides [2]. During their transposition, helitrons can capture, amplify and disperse complete genes and gene fragments by a yet unknown mechanism [11–16], which seems to be an important tool for the evolution of new helitrons [17]. Thus, they contribute to a breakdown in genetic colinearity, as previously described in maize haplotypes. Helitrons play an important role in the creation of new proteins via exon shuffling and gene duplication. According to Yang et al.[14], most of the genes captured by helitrons in maize are subjected to genetic drift, 4% of them to purifying selection and 4% of them to adaptive selection, which suggests that there is a benefit for helitron-carrying species. Helitrons have highly variable lengths (ranging from 202 bp to 35.9 kb in maize) and abundance in eukaryotic genomes. In the fruit fly *Drosophila melanogaster*, helitrons account for 1 to 5% of the total size of the genome [3], and in mammals such as *Myotis lucifugus*, they account for 3% [13]. In plants, the contribution of helitrons to the total genome size is variable. In *A. thaliana*, helitrons account for more than 2% [2], whereas in *Oryza* the estimations vary from 0.03 in *O.brachyantha*[18] to 4% in *O.sativa*[19]. In maize (where they have been better characterized [9, 11, 12, 20]) the latest analysis reports the presence of 31.233 helitron copies accounting for 6.6 % of the B73 reference genome [19]. In fungi, helitron-like sequences have been identified *in silico* in the genomes of species belonging to the phylum Ascomycetes (such as *Aspergillus nidulans, Chaetomium globosum*, *Fusarium oxysporum*, and *Tolypocladium inflatum*), as well as in the zygomycete *Rhizopus oryzae* and the phylum Basidiomycetes (such as *Phanerochaete chrysosporium, Coprinopsis cinerea, Ceriporiopsis subvermispora, Laccaria bicolor* and *Puccinia graminis*) [3, 21–27]. However, these studies only reported on the presence of helitrons and their densities; their structural features were not analyzed. Thus, we lack a general picture of the structure of fungal helitrons, as well as an understanding of their role in gene capture and their broader genomic impact. *Pleurotus ostreatus* is a white rot basidiomycete that is widely used as a model organism. Recently, the genome of the dikaryotic strain N001 of *P. ostreatus* (which is approximately 34 Mb and organized in 11 chromosomes), was comprehensively analyzed. The genome sequences of the monokaryotic parentals PC9 and PC15 are available [28]. Sequence analysis of both *P. ostreatus* strains revealed the presence of helitrons in strain-specific genomic locations, as described for different maize haplotypes. The presence of helitrons led to a lack of synteny between both haplotypes and the occurrence of important rearrangements. With the aim of uncovering new insights into the role of helitrons in the *P. ostreatus* genome as well as the genomes of other ascomycetes and basidiomycetes, we report on the following: i) their structural features and functional domains, ii) their abundance and occurrence in PC9 and PC15 genomes, and iii) their ability to capture, create and express new genes. Finally, we investigate the helitron landscape in *P. ostreatus* and other sequenced fungi to understand their origins and evolution in the fungal kingdom.

## Results

### *Pleurotus ostreatus*helitrons

**Figure 1:**
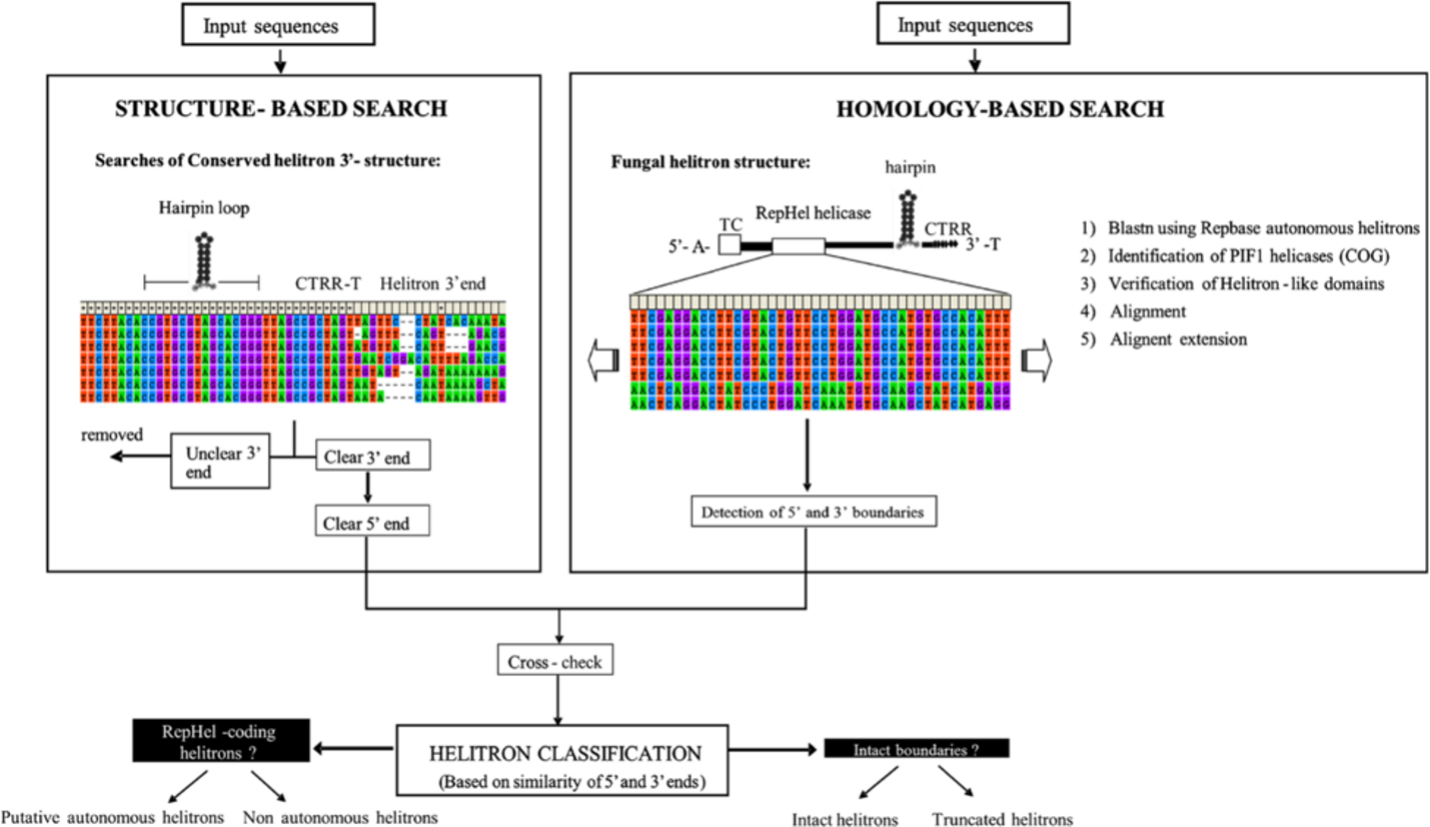
**Pipeline for helitron identification in the** ***P. ostreatus*** **PC9 and PC15 genomes.**

**Figure 2:**
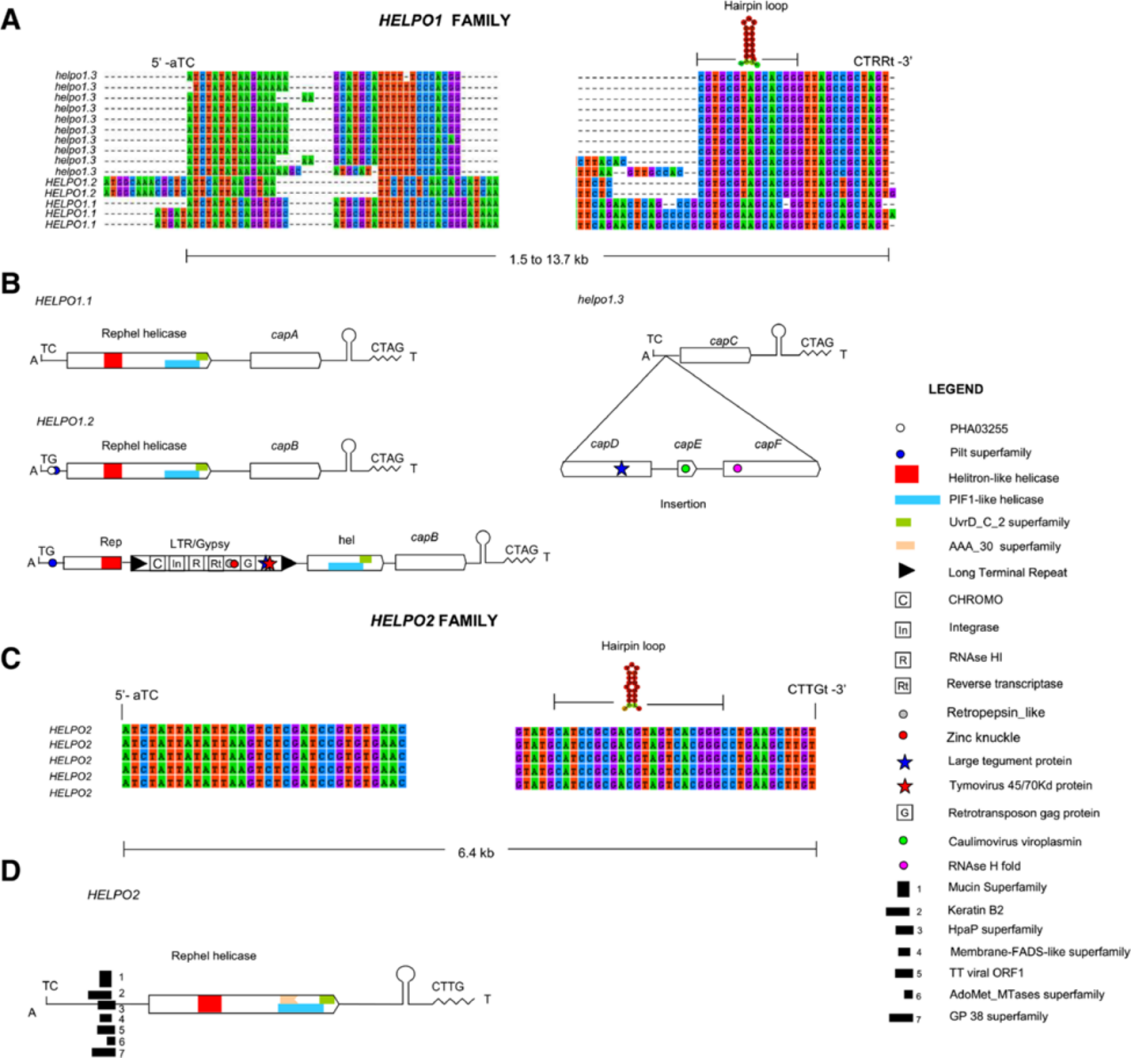
**Structural and enzymatic features of the** ***P. ostreatus*** **helitron families.** Alignments of the 5′ and 3′ boundaries of the helitron families HELPO1 **(A)** and HELPO2 **(C)**. Schematic representation of the structural hallmarks, coding features and conserved domains (CDD, cutoff E-value <0.01) of the different elements belonging to the HELPO1 **(B)** and HELPO2 **(D)** families.

We designed a pipeline for helitron identification in *P. ostreatus* (Figure 1) starting with a structure-based approach using HelSearch. This approach yielded 11 and 9 putative helitron families in the PC15 and PC9 genomes, respectively (Additional file 1: Table S1). Our subsequent homology-based approach uncovered another putative helitron family that could not be detected by the first method. After a manual curation of the alignments and the removal of false positives, we obtained two verified helitron families named HELPO1 and HELPO2. Both families contain most of the structural and enzymatic features described earlier in plant/animal helitrons such as AT insertion specificity, T[C/G]-5′ and CTRR-3′ ends (CTTG in the case of HELPO2), the presence of a subterminal palindromic hairpin, and a rolling-circle replication initiator as well as a helicase domain in a common ORF (Figure 2). Based on the similarity of the 5′ and 3′ boundaries (see Materials and Methods), helitrons of the HELPO1 family can be further classified into three subfamilies: HELPO1.1, HELPO1.2 and HELPO1.3, with elements ranging from 1.5 to 13.7 kb length (Figure 2.A). The similarity of their 5′ and 3′ helitron ends indicates that the HELPO1.2 and HELPO1.3 subfamilies are more closely related, while HELPO1.1 is more distant (data not shown). HELPO2 contained elements varying from 3.9 to 10 kb in length. Both the HELPO1 and HELPO2 families contain putative autonomous elements, however, the HELPO1 family is the only one carrying intact non-autonomous copies, all of them belonging to subfamily HELPO1.3 (Figure 2). The flanking regions of the helitron insertion sites (50 bp) are AT-rich. In fact, these regions show an AT content of 57%, while the helitrons’ AT content is similar to that of the whole genome (49%). The putative autonomous elements of the HELPO1 and HELPO2 families carry an ORF encoding a RepHel helicase of approximately 1400 aa. The protein contains three motifs defining the rep domain [3] as well as six conserved motifs present in members of the SF1 helicase superfamily described in other helitrons (Additional file 2: Figure S1) [13, 29] and necessary for replication and DNA unwinding. Using a maximum likelihood approach, we clustered the RepHel helicases into three groups (Additional file 2: Figure S1), where the *HELPO1* and *HELPO2* proteins were grouped separately. Interestingly, the third group lacks the rolling-circle replication initiator but ferries some of the helicase domains. Apparently, these helicases do not belong to a specific helitron family. It should be pointed out that putative HELPO1 and HELPO2 autonomous elements share about 60-70% similarity to Helitron 1_SLL_1p of *Serpula lacrymans*[30] and Helitron2_Ppa_1p of *Physcomytrella*[31], but only in the regions corresponding to the Helitron helicase-like domain (Pfam PF14214) and the PIF1-like helicase domain (Pfam PF05970).

### Helitron abundance in the *P. ostreatus*PC15 and PC9 homologous genomes

**Figure 3:**
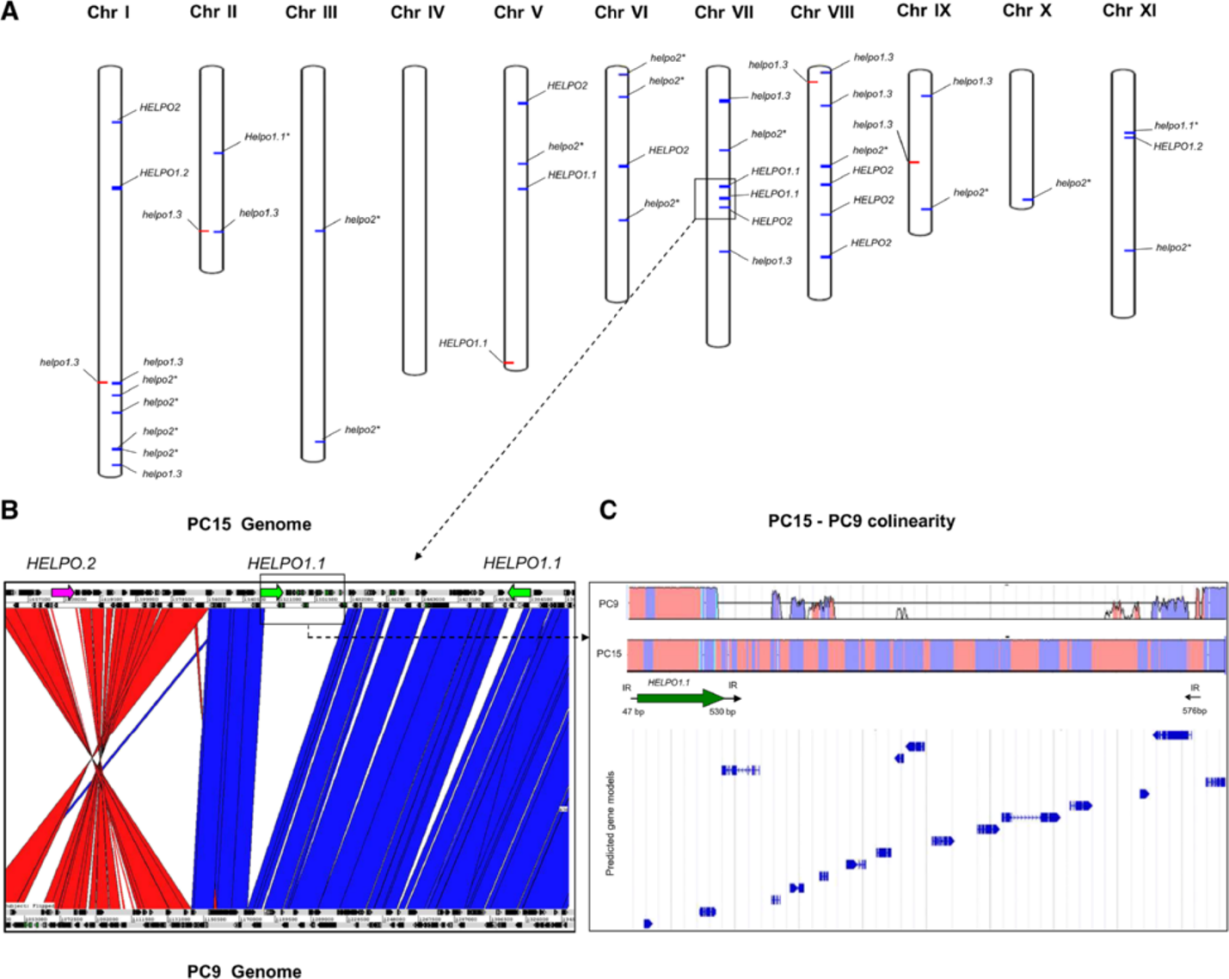
**Helitrons break the synteny between the** ***P. ostreatus*** **PC15 and PC9 genomes.** The distribution of helitrons in the chromosomes of the dikaryotic strain N001 is shown in **A** (PC15 elements are shown in blue and in PC9 elements are shown in red). Truncated elements are marked with a ‘*’. An ACT [32] comparison of the squared region between PC15 and PC9 is shown in **B**. The lack of gene colinearity between PC9 and PC15 in the squared region of chromosomeVII is shown in **C** (coordinates: 1,528,715-1,479,715). In the synteny plot, coding regions are represented in purple, and inter-genic regions in pink. Arrows labeled IR represent the inverted repeats found in a 37.2 kb region duplicated in PC15 and absent in PC9 genome. Blue arrows underneath synteny plot represent predicted genes.

A total of 37 validated helitrons in the HELPO1 and HELPO2 families were detected in the PC15 strain (Figure 3A, Table 1), accounting for 0.35% of the total genome size. Among these helitrons, 19 were intact elements, 11 out of the 19 were full-length putative autonomous elements, and the remaining elements were truncated copies. In the PC9 genome, 10 helitrons accounting for 0.05% of its genome were found, of which only five could be mapped to the corresponding PC15 scaffolds (Figure 3A). Five elements showed intact 5′ and 3′ boundaries, one was putative autonomous (*HELPO1.1*), and the rest were truncated elements.

**Table 1:** **Summary of the helitron characteristics in the** ***P. ostreatus*** **dikaryotic strain N001**

Name/ID	Genome	Scaffold	Start (bp)	End (bp)	Orientation	Size (Kbp)	Autonomous	Captured genes	Intact	RPKM
*HELPO1.1*	PC15	5	1418337	1425442	-	7,1	*	*capA2*	*	38,64
*HELPO1.1*	PC15	7	1528175	1528175	-	7,2	*	*capA*	*	13,64
*HELPO1.1*	PC15	7	1387822	1395008	+	7,2	*	*capA*	*	16,05
*HELPO1.1*	PC15	11	702113	708712	+	6,6	*	*capA*		0,00
*HELPO1.2*	PC15	1	1404200	1417948	+	13,7	*	*capB*	*	1,38
*HELPO1.2*	PC15	11	756984	764123	+	7,1	*	*capB*	*	2,41
*helpo1.3*	PC15	1	3742549	3754728	+	12,2		*capC, CapD, capE, CapF*		0,65
*helpo1.3*	PC15	1	4537580	4539124	-	1,5		*capC*	*	5,43
*helpo1.3*	PC15	2	1934871	1936414	-	1,5		*capC*	*	7,69
*helpo1.3*	PC15	7	380915	382459	+	1,5		*capC*	*	6,33
*helpo1.3*	PC15	7	2181803	2183334	+	1,5		*capC*	*	5,04
*helpo1.3*	PC15	8	26555	28057	-	1,5		*capC*	*	3,29
*helpo1.3*	PC15	8	423643	425136	-	1,5		*capC*	*	5,45
*helpo1.3*	PC15	9	258831	261375	-	1,5		*capC*	*	6,23
*HELPO2*	PC15	1	619761	62150	+	6,4	*		*	0,18
*HELPO2*	PC15	5	387607	398218	-	10,6	*			0,14
*HELPO2*	PC15	6	1150050	1156438	-	6,4	*		*	0,20
*HELPO2*	PC15	7	1635256	1641644	-	6,4	*		*	0,19
*HELPO2*	PC15	8	1367660	1374048	+	6,4	*		*	0,19
*HELPO2*	PC15	8	2234922	2241310	-	6,4	*		*	0,18
*HELPO2*	PC15	8	1722302	1726240	+	3,9	*			0,03
*HELPO2*	PC15	11	2114358	2115721	+	1,4	*			0,00
154430	PC15	2	1672984	1678495	-	5,5	//			0,00
1035322	PC15	2	1778564	1779100	-	0,5	//			0,00
1044620	PC15	7	2753773	2756802	+	3,0	//			0,00
1078941	PC15	8	2474562	2480319	+	5,8	//			0,00
1078947	PC15	8	2505293	2510116	-	4,8	//			0,00
1079561	PC15	10	1356533	1360752	-	4,2	*			1,00
*HELPO1.1*	PC9	115	1	7176	-	7,2	*	*capA*	*	17,59
*HELPO1.1*	PC9	91	1	560	+	0,6		*capA*		0,0
*HELPO1.2*	PC9	366	1	3061	+	3,1	*	*		0,25
*helpo1.3*	PC9	7	1079490	1082511	+	3,0		*		2,62
*helpo1.3*	PC9	44	2611	4188	-	1,6		*	*	6,41
*helpo1.3*	PC9	142	1	533	+	0,5		*		5,37
*helpo1.3*	PC9	360	2475	3115	+	0,7		*		5,65
*helpo1.3*	PC9	375	1	440	-	0,4		*		3,64
*helpo1.3*	PC9	478	373	1917	-	1,5			*	9,27
*HELPO2*	PC9	440	1	2592	-	2,6	//	*		0,00
48294	PC9	2	447393	449295	+	1,9	//			3,07
*51890*	PC9	3	2765812	2767353	+	1,5	//			0,00
52065	PC9	3	5913	9386	+	3,5	//			0,00

A transcriptional analysis was performed using the RPKM method and the RNA-seq reads were mapped to the PC15 and PC9 homologous genomes. Symbol *indicates that the element fits the description shown in the header, while // indicates the contrary.

Helitron length polymorphisms were observed in some of the elements. Members of the HELPO1.2 subfamily showed two elements of different lengths. The shortest element (7.1 kb) was located on chromosome XI, and the largest (13.3 kb) was located on chromosome I. The HELPO1.3 subfamily was the only subfamily with non-autonomous elements at identical positions in both genomes. In this sense, it should be noted that the large *helpo1.3* copy appeared as an allele of the short copy on chromosome I. Copies of the short *helpo1.3* copy were also found on chromosome II. In PC15, helitrons were found in ten out of eleven chromosomes. Seven chromosomes carried helitrons from both families, while three (chromosomes II, VI and X) carried helitrons from only a single family. Chromosomes I, VII and VIII carried the highest number of helitrons. Clusters of helitrons were present in the regions of chromosomes I and VII (Figure 3A) showing 50% GC content. Breaks in gene colinearity between PC15 and PC9 were observed in 66% of the helitron containing regions (except in the genome regions described above), as shown in Figure 3. The analysis of 44 regions of 50 kb adjacent to HELPO1 and HELPO2 helitrons (see Additional file 3) revealed that the frequency of colinearity breaks in these regions was 1.86 every 50 kb, while the frequency in the whole chromosome I was 1.25 breaks every 50 kb. According to our results, 40% of the PC9 missing counterparts (non-homologous regions) were present in a different location, while 22% corresponded to other transposable elements, mainly LTR/Gypsy, DNA/PIF-Harbinger and DNA/CMC-EnSpm. In chromosome VII, the two *HELPO1.1* copies showed 99.7% similarity. One of the copies was inserted into the left 576-bp inverted repeat found in a 37.2-kb region present on a chromosome of PC15 but was absent in the PC9 genome (Figure 3C). This region was also found close to the telomere in chromosome XI of PC15 and carried 14 predicted genes.

### Helitron captured genes

The helitrons of the HELPO1 family show a high tendency for gene acquisition/creation, as every intact copy carried from one to four gene-like sequences (Figure 2B). By contrast, members of HELPO2 only contained the RepHel helicase. In PC15, putative autonomous elements of the HELPO1 family carried from one to four captured genes (*cap*) downstream of the RepHel helicase (Figure 2B). The captured genes of the HELPO1.1 subfamily were named *capA*, those from HELPO1.2 were named *capB*, and those from HELPO1.3 were named *capC*, *capD*, *capE* and *capF* (Figure 2B). The captured gene of the HELPO1.1 copy on chromosome V was named *capA2* instead *of capA* due to its low similarity to the other *capA* genes (45%, see Additional file 4: Table S3) carried by the helitrons on chromosome VII. Chromosome XI harbors a *capB* gene in a *HELPO1.2* element. Interestingly, an extra copy of the HELPO1.2 subfamily carrying (apart from the *capB* gene) an LTR/Gypsy retrotransposon (>70% similarity of nucleotide sequence to Gypsy-8_CCO-I of *C. cinerea*, Repbase) was found on chromosome I. The LTR/Gypsy retrotransposon was inserted in the second reverse reading frame, breaking the RepHel helicase ORF (Figure 2B). Several copies of this retroelement were found in chromosomes I, III, V, IX and XI of PC15. The genes carried by *HELPO1* helitrons can be classified based on their conserved domains as retrotransposon/viral genes or as genes of unknown function.

#### Retrotransposon/viral genes

**Figure 4:**
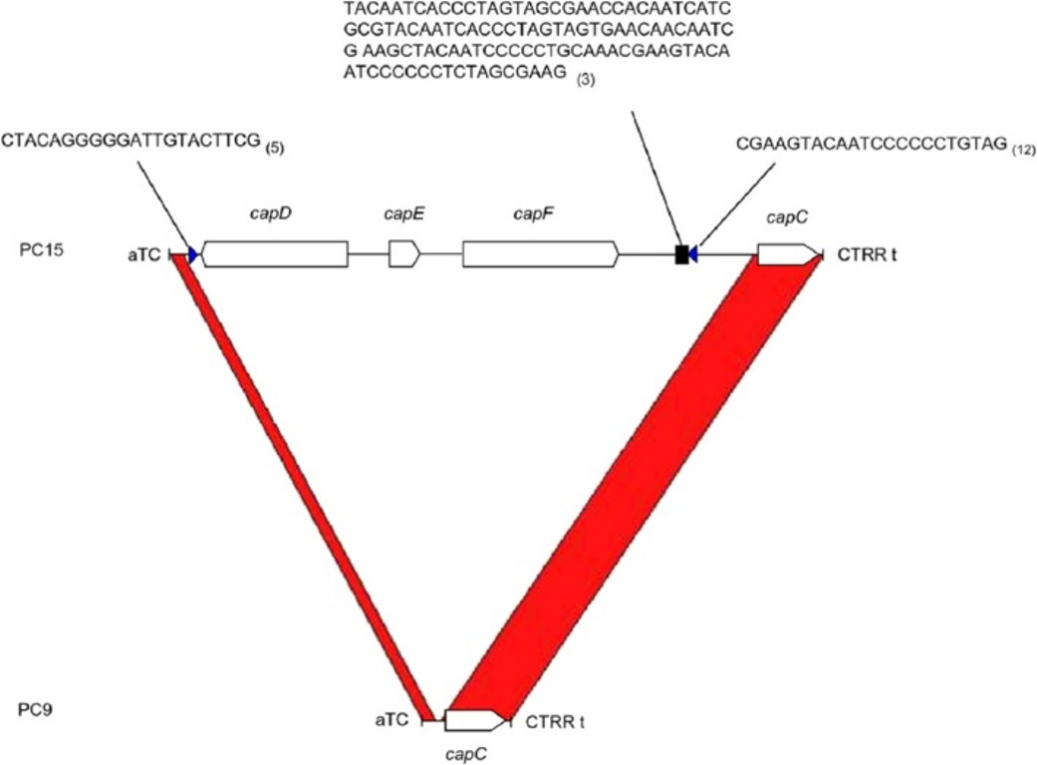
**Helitron length polymorphisms in allelic copies of the HELPO1.3 subfamily.** Regions in red are highly conserved. Blue triangles represent inverted repeats, and the black square represents a satellite sequence (the number of repeats is shown in parentheses). Empty arrows represent predicted ORFs.

An analysis of the conserved domains showed significant hits (CDD, cutoff E-value <0.01) in a *HELPO1.2* copy harboring LTR/Gypsy and in a *helpo1.3* copy, both present on chromosome 1 (Figure 2B, Table 1). The *HELPO1.2* copy on chromosome I carried viral and retrotransposon domains in addition to helitron motifs (Figure 2, Additional file 5: Dataset S1). BLASTN searches performed on PC15 filtered model genes using intact helitrons as queries showed that this *HELPO1.2* was the only helitron harboring plant and animal re-arranged retroviral genes shuttled by a retroelement. The largest *helpo1.3* copy on chromosome I was 10.7 kb longer than the mean of the lengths of the other *helpo1.3* copies in the *P. ostreatus* genome (12.2 kb vs. 1.5 kb, Figure 2B and Figure 4), and it bore a small EST without a predicted gene model (the *capC* gene) as well as three predicted genes (*capD*, *capE* and *capF*). The *capD* gene contains a domain present in the large tegument protein UL36 of the herpes virus (PHA03247), *capE* carries a Caulimovirus viroplasmin (pfam01693), and *capF* carries a predicted nuclease (RNAse H L fold, COG4328). All of the *cap* genes described above are exclusively captured by helitrons and do not have additional copies outside helitron boundaries.

#### Genes of unknown function

*capA*, *capA2*, *capB* and *capC,* did not bear conserved domains. A BLASTX query of the entire MycoCosm database (cutoff E-value <10^−10^) revealed that the *capA*, *capA2* and *capC* genes were novel *P. ostreatus*-specific fungal genes, while *capB* yielded significant hits for proteins of unknown function that are present in a few species of Basidiomycetes: *Armillaria mellea* (ID: 8292), *Dendrothele bispora* (ID: 811331), *Fibulorhizoctonia* sp*.* (ID: 941557), *Schizophyllum commune* Loenen (ID: 271731), and *Suillus brevipes* (ID: 956931). With the exception of *A. mellea* (because the gene was at the end of the scaffold), all of the species carried the RepHel helicase in the same orientation as the *P. ostreatus* helitron *HELPO1.2*, as evidence of the patchy distribution of this helitron subfamily in the phylum Basidiomycota.

No hits (cutoff E-value <10^−5^) for any promoter transcription factor motifs were found in BLAST searches against fungal (MycoCosm) and Viral (viroBlast) databases.

### Transcription

**Figure 5:**
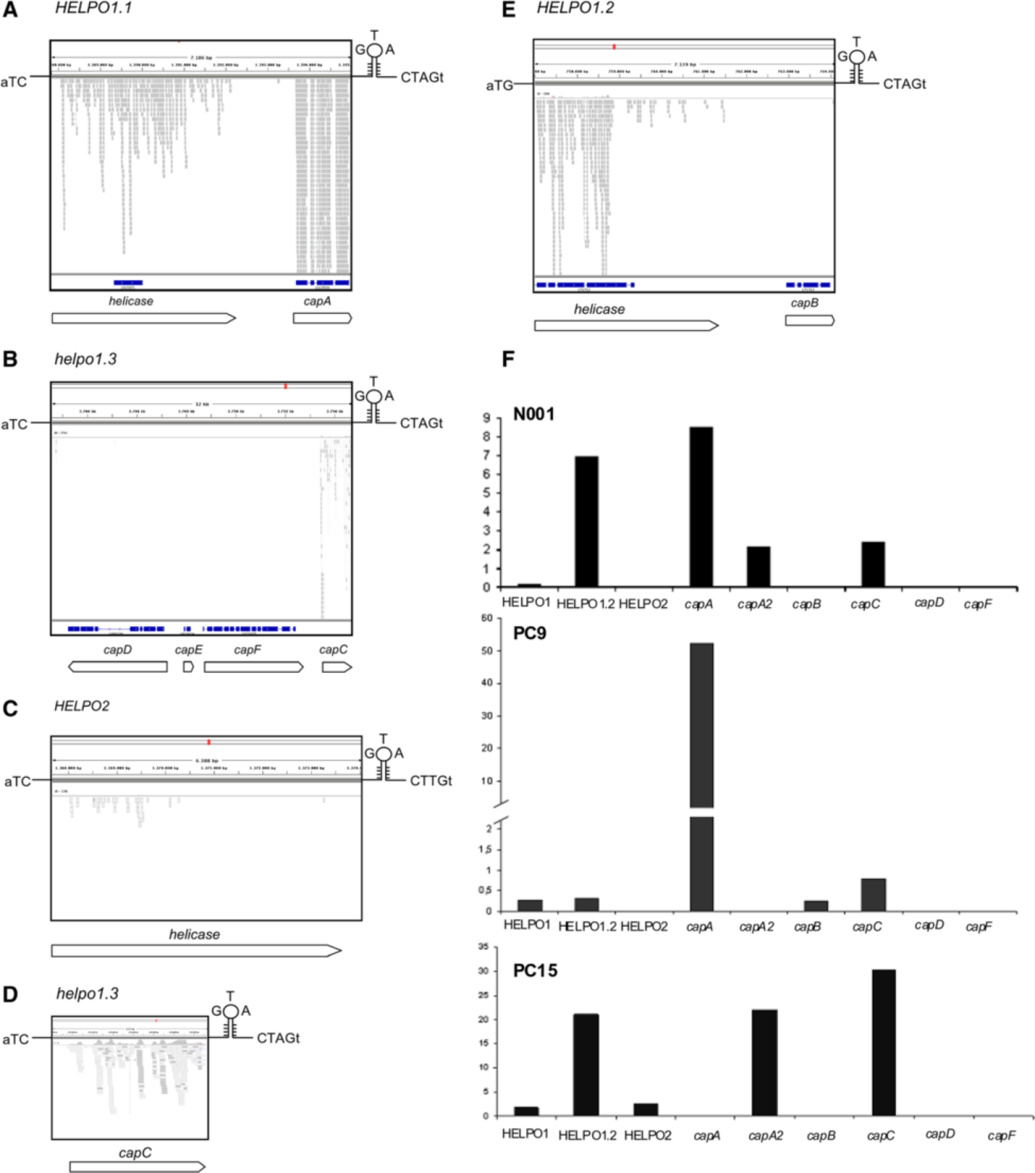
**Transcriptional profiles of helitron-specific helicases and captured genes.** Five representative RNA-seq profiles of the helitron families and subfamilies **(A** **to** **E)**. The gene models predicted by JGI are shown in blue. Empty arrows represent manually annotated genes. The expression of the N00, PC9 and PC15 RepHel helicases and captured genes by RT-qPCR is shown in **F**. The Y axis of F represents the arbitrary units (RQ ) relative to the expression of the reference gene *pep*.

The transcriptional profiles of 30 helitrons and 10 truncated RepHel helicases from the *P. ostreatus* PC15 and PC9 genomes were investigated in solid SMY cultures using RNA-seq in the dikaryotic strain N001 (Figure 5, Table 1). An analysis of RNA-seq reads using IGV [33] yielded different profiles for the members of different families and subfamilies (Figure 5). In most cases, the RNA-seq reads did not fit with the gene models predicted by the JGI, although we also found RNA-seq reads that mapped to regions with no annotated models, for example, *capC.* Helitrons in the HELPO1 family showed higher levels of transcription (based on the RPKM values of the entire helitron, including the RepHel helicase and the captured genes), in comparison with the elements belonging to the HELPO2 family. The truncated PIF1 helicases showed no transcriptional activity, with the exception of helicase ID 1079561 (on chromosome X). The HELPO1.1 subfamily displayed very high levels of expression (up to 38.64 RPKM) compared with the HELPO1.3 (maximum of 7.69 RPKM) and HELPO1.2 members (maximum of 2.41 RPKM). RT-qPCR experiments were performed using mRNA from the strains PC9, PC15 and N001 grown in submerged cultures to analyze the expression of the RepHel helicases and captured genes independently. For RepHel helicases, similar relative profiles were observed in the three strains, although the ranges of the transcriptional levels were different (Figure 5F). The RepHel helicase of HELPO1.2 was frequently the most highly expressed (0.31, 20.9 and 6.9 RQs in PC9, PC15 and N001, respectively). HELPO1.1 RepHel showed much lower expression levels (0.25, 1.8 and 0.2 RQs in PC9, PC15 and N001, respectively) and HELPO2 showed no expression in N001 and PC9 (0, 2.6 and 0 RQs in PC9, PC15 and N001, respectively). Virus-like captured genes carried by LTR/Gypsy did not show transcription in any strain, and genes of unknown function, such as *capA, capA2*, *capB* and *capC,* showed a strain-specific expression profile. RT-qPCR experiments performed with PC9 showed that *capA* was the most highly expressed gene (52.3 RQs) whereas much lower (*capB* and *capC*) or no transcription (*capA2*) was observed for the rest of these genes. In PC15, the highest expression values corresponded to *capC* and *capA2* (30.4 and 22.1 RQs). In N001, c*apA* was the most highly expressed gene, followed by *capC* and *capA2* (8.51, 2.4 and 2.2 RQs) (Figure 5F). Clear differences were observed between the *capA and capA2* transcription profiles using RT-qPCR. Because the primers were designed to amplify more than one gene with the exception of *capA2*, *capD* and *capF* (Additional file 6: Table S4), the transcription levels obtained were the result of the contribution of every RepHel helicase and captured gene from each helitron family.

### Differential expansion of the helitron-specific helicases in other fungi

TBLASTN homology-based searches were carried out on the entire MycoCosm database (as of January 2014) using the Helitron helicase-like (PF14214, 182 aa) and PIF1-like helicase (PF05970, 362 aa) domains as queries. The search yielded 1311 and 1645 significant hits in 149 genomes (cutoff E-value <10^−5^) to the Helitron helicase-like and PIF1-like helicase domains, respectively. The results were used to analyze the expansion of helitron-specific RepHel helicases in fungal phyla. We found a clear difference in the occurrence of helitron-like helicases in the Ascomycetes and Basidiomycetes classes. While 87% of the genomes of the basidiomycetes analyzed contained RepHel proteins, only 30% of the ascomycetes contained RepHel proteins. This difference is even more striking when we consider that the ascomycetes group comprised a larger number of analyzed genomes. Interestingly, the correlation of the presence of both domains was very high (r = 0.91) in fungi.

### Phylogenetic reconstruction of eukaryotic RepHel helicases

**Figure 6:**
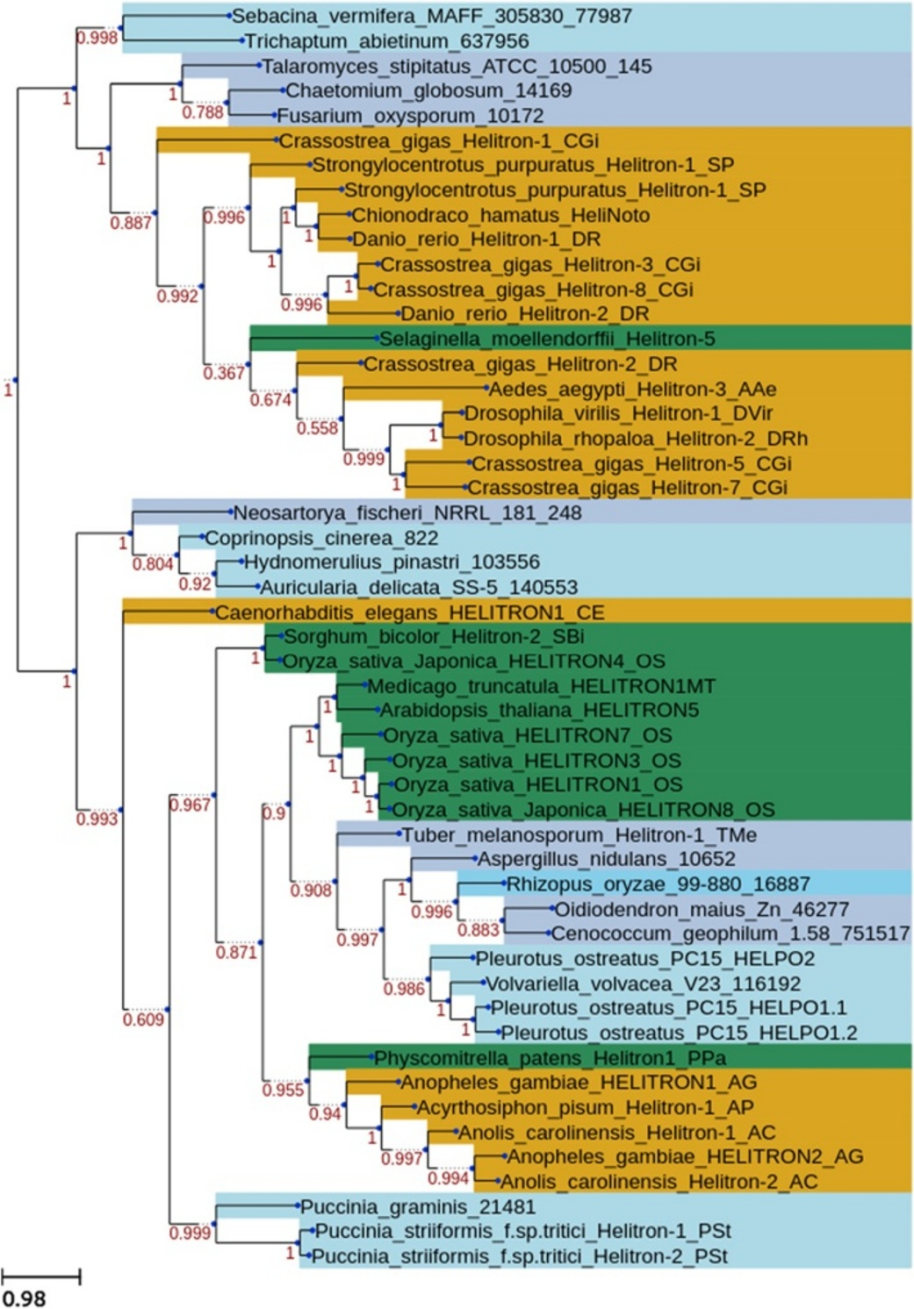
**Phylogenetic reconstruction of the eukaryotic Pif1-like helicase domain.** Green represents helitrons from the Plant kingdom, yellow from the Animal kingdom, and blue from the Fungal kingdom. Light blue represents the phylum Basidiomycota and dark blue represents the phylum Ascomycota.

To investigate the evolutionary relationships of the fungal helitrons identified as well as those from other eukaryotic genomes, we reconstructed molecular phylogenies of the PIF1-like helicase and Helitron helicase-like domains (see Materials and Methods). An initial dataset containing 2175 PIF1-like helicases from 284 fungal genomes (JGI filtered models) and 213 putative autonomous elements obtained from Repbase (including plants, animals and fungi) was used to uncover new insights into the helitron distribution in the eukaryotic domain. A total of 672 sequences bore the PIF1-like helicase domain, 416 carried the Helitron helicase-like domain, and 125 sequences displayed both domains. After removing duplicated copies, the remaining sequences were used for phylogenetic analyses. The functional domains were extracted from the sequences and aligned using custom Python scripts. Both analyses (Figure 6 and Additional file 7: Figure S2) depicted a similar scenario - fungal helitrons were not monophyletic, but rather they appeared in at least four different clades interspersed among metazoan and plant helitrons. In addition, within each fungal clade, the different fungal phyla (e.g., ascomycetes, basidiomycetes) appeared mixed.

## Discussion

Previous studies have shown that helitron transposons are widespread in eukaryotic genomes [2, 13, 34, 35]. Their structural and enzymatic features have been analyzed in depth in plants and animals using computational analyses, uncovering a canonical structure that is widely conserved among the elements in both kingdoms. Several tools and pipelines have been published for analyzing helitrons in a diverse range of eukaryotic genomes [20, 29, 36]. These approaches rely on either homology-based searches of previously known helitrons or structure-based searches of unique helitron features such as the conserved 3′- terminus. However, fungal helitron-like sequences can lack intact boundaries [3]. This characteristic impedes helitron identification using structure-based searches. In *P. ostreatus*, we show that both the structural and coding features (the Rep and Helicase domains) are present and highly conserved with the structural and coding features present in other helitrons in different kingdoms. Nevertheless, the elements of the HELPO2 family containing a slight variation in the 3′-terminus are not distinguishable using HelSearch. This situation necessitates combining homology-based searches, structure-based approaches and manual curation for fungal helitron searches, as described in this paper. In terms of relative abundance, the helitron content of *P. ostreatus* is similar to that of other basidiomycetes (0 to 0.5% of their genome size) [27]. We found that genome assemblies of poor quality critically impacted helitron searches, leading to underestimates of the helitron content. The *P. ostreatus* PC15 genome sequence was assembled into 11 scaffolds, which fit with the 11 known linkage groups [37]. However, the PC15 scaffolds were not used as templates for the PC9 assembly because our goal was to analyze the effect of helitrons in breaking synteny and the consequences of hemizygous regions with respect to *P. ostreatus* mushroom yield and enzyme expression. Thus, we found the estimation of helitron abundance for the PC15 genome to be more accurate than the PC9 genome because PC9 is assembled into 572 scaffolds, most of which are very small in size. *P. ostreatus* helitrons insert precisely between A and T nucleotides; they often land in AT-rich genome regions occupied by other helitrons [14]. In *P. ostreatus*, approximately half of the helitrons were found in retrotransposon-rich regions (data not shown). This phenomenon is more pronounced in the *helpo1.3* and *HELPO2* elements because they are more abundant. A high percentage of HELPO1 helitrons were putative autonomous elements carrying captured genes inside their boundaries compared with HELPO2. The similarity between the elements belonging to different families and subfamilies (approximately 40% between HELPO1 and HELPO2, and approximately 60% between HELPO1.1 and HELPO1.2, Additional file 8: Table S2) strongly suggests that helitron vertical diversification has occurred. However, recent amplification events are not excluded because both the HELPO1 and HELPO2 families contain young elements (i.e., HELPO1.3 and HELPO2 display elements with 99-100% similarity). Notably, the short copy of *helpo1.3* (1.5 kb) occurs frequently in the *Pleurotus* genome compared with the large one (15 kb, present only once). The long copy contains internal complementary repeats flanking capD and capF genes. These sequences may promote an intrachromosomal rearrangement leading to the formation of a loop that contains the captured lost genes *capD*, *capE* and *capF*. The short copy of *helpo1.3* would then bear only *capC,* which is later amplified. In the *Pleurotus* genome, the mobilization of LTR/Gypsy elements and their insertion into helitrons creates chimeric elements. For example, a LTR/Gypsy element containing animal and plant viral sequences present in several Basidiomycetes genomes was found in an opposite orientation breaking the RepHel helicase ORF of a *HELPO.1.2* element in *P.ostreatus* PC15. This finding supports an insertion rather than a capture of the LTR/Gypsy element by a helitron. This result greatly differs from that found in plants and animals, where helitrons frequently capture gene fragments from their hosts [13, 14, 34]. Previous studies by [38] found that chimeric elements formed by helitrons and other TEs are rare in eukaryotic genomes.

### Helitron-mediated amplification and expression of captured genes

*Pleurotus* helitrons contain a subterminal hairpin and a well-conserved 3′-CT[A/T)G end, and they do not generate target site duplications in agreement to what was previously described for other eukaryotes [2, 14]. The conservation of the 3′-end structure in helitrons from highly divergent species (i.e., fungi and plants) suggests that the 3′-end structure plays an important role in transposition [7]. Earlier studies have hypothesized that this structure could serve as a terminator transposition signal. In this sense, the proposed read-through-model-1 (RTM1) [7] suggests that a malfunction of this RC terminator may lead to the acquisition of genes or gene fragments adjacent to the 3′ helitron end. The location of captured genes downstream of the RepHel helicase (i.e., *capA* and *capB*) fits with the RTM1 model of gene capturing through new 3′-end acquisition, although there were no clear intermediate RC terminators representing ancient helitron-ends. This could be due to the deletion of the 3′ terminus during transposition or sequence degeneration. In fact, the RC terminator in the new transposon is formed *de novo* by a terminator-like signal in the surrounding location, as described in the capture of a fragment of the xanthine α-ketoglutarate-dependent dioxygenase gene by a non-autonomous *Helitron-N1_AN* from *A. nidulans*[21]. In plants and animals, helitrons contain genes captured from their hosts [13, 14, 34]. In *P. ostreatus*, the fact that there are very few significant BLAST hits in databases using *capB* as a query in addition to the absence of hits using the other *cap* genes as queries indicates that *cap genes* are either novel structures created by shuffling DNA sequences from diverse origins or the result of a full gene capture in a host other than fungi whose sequence is still not available. The bias found in the gene capturing frequencies of the HELPO1 (high frequency) and HELPO2 (no captured genes) families, as well as the scarce and patchy distribution of some of these genes in fungal phylogeny, gives strength to the hypothesis of an ancient capture in a previous host. The architecture of the non-autonomous copy of the HELPO1.3 family that carries four predicted genes (Figure 2, Figure 4) fits better with a filler DNA model in which the captured regions are acquired by the machinery responsible for the non-homologous repair of double-stranded DNA breaks [3]. A similar integration mechanism was described for viral genomes [39]. Recently, due to the increasing number of whole genome sequencing projects and bioinformatics analysis tools available, a large body of literature has been reported regarding virus integration into eukaryotic genomes (endogenous viral elements, EVE) and their roles in their hosts [40, 41]. The presence of virus-related domains within an LTR/Gypsy element in a *HELPO1.2* copy as well as the occurrence of virus domains in HELPO2 elements suggests that viruses may have participated in the horizontal transfer of these elements from an anonymous ancestor to basidiomycete fungi. The lack of captured genes in the HELPO2 family, along with the above mentioned fact, suggests that fungal helitrons are less likely to capture genes and/or gene fragments than plant and animal helitrons. In fact, none of the intact elements showed any evidence of carrying *P. ostreatus* gene fragments. The captured genes *capD* and *capE* of the *helpo1.3* element also contain animal (the large tegument proteins UL36 of the herpes virus (PHA03247)) and plant (Caulimovirus viroplasmin (PF01693)) endogenized viral sequences. Some researchers have described the occurrence of footprints resulting from EVE integration into host genomes mediated by the retrotransposon enzyme machinery (for review, see [41]). We have not identified any footprint in the *P. ostreatus* genome resulting from gene capture. However, the captured gene *capF* contains a domain (COG4328) that was recently classified *in silico* as a putative transposase. It is possible that this transposase, together with the TIR elements flanking the *capD* and *capF* genes, promoted a chromosome loop in the large copy of *helpo1.3*, resulting in the short *helpo1.3* copy*.* With the exception of HELPO1.3, the HELPO1 and HELPO2 families contain putative autonomous elements containing three motifs that define the catalytic core [3] as well as the helicase domain. Although fungal RepHel helicases are often described to be intronless [3], the RNA-seq profiles of the *P. ostreatus* strain N001 revealed the presence of introns in the RepHel genes of the *HELPO1.1* and *HELPO1.2* elements (Figure 5). We did not find any of the previously described domains in the RepHel ORF such as the replication protein A (RPA) found in plant helitrons [2] and occasionally in animals [35], the zinc fingers present in cnidarian, insect, fish, frog, reptile and mammalian helitrons, or the apurinic (EN) and cysteine protease (CPR) found in cnidarian, fish and frog helitrons. In contrast, a set of conserved domains from viruses, bacteria and eukaryotes never found before in helitrons (Figure 2, Additional file 5: Dataset S1) were present in *P. ostreatus.* The similarity between the RepHel proteins in HELPO1.1 and HELPO1.2 (68.5%) indicates their importance for helitron-specific functions. The similarities between *capA, capA2* and *capB* (approximately 45%) suggest that a functional divergence could have occurred, leading to the maintenance (or suppression) of their activities that conferred a possible advantage for the host genome. In this sense, the RT-qPCR experiments showed the highest levels of expression of the *capA* and *capA2* genes in the PC9 and PC15 strains and lower expression levels of *capB*. It should be mentioned that the *capA* gene carried by the *HELPO1.1* elements maps to chromosome VII in a region containing a QTL for earliness and mushroom yield in the dikaryotic strain N001 (R^2^ = 32.07). We are currently studying in dikaryons derived from monokaryotic progeny of N001 to find out whether *capA* transcripts have any influence on earliness and mushroom yield.

### Phylogenetic reconstruction of Rephel helicases

The helitron helicase-like and Pif1-like helicase domains are present in the putative autonomous elements of every species and are under selective pressure because they are essential for helitron transposition. Thus, these domains retain conserved motifs that can be used to infer the phylogenetic relationships between the helitrons of different organisms. This feature is relevant considering the high variability present within helitron boundaries driven by their ability to capture and reshuffle gene fragments from their hosts. Our phylogenetic analysis revealed a clear polyphyletic origin of these domains, suggesting that horizontal gene transfer played a role in shaping the current distribution of helitrons in extant eukaryotic genomes. Nevertheless, the direction and order of these events cannot be properly assessed given our current sample size. The differential expansion of RepHel helicases in ascomycetes and basidiomycetes, along with the presence of viral domains within helitron boundaries gives strength to the hypothesis of horizontal transfer. In fact, viruses have been proven to be vectors of horizontal transfer of other TEs between eukaryotic hosts sharing viral pathogens [42–44]. An important point to emphasize is that, in addition to plant and animal viruses, bacterial and eukaryotic domains were also found to be integrated into *Pleurotus* helitrons. Previous genomics analyses have shown that HGT could play a more important role in fungal evolution than originally thought [45]. Previous results from our laboratory described a bipartite structure similar to that of the *A. terreus* genome located in a subtelomeric region in *P. ostreatus*[46]. This suggests a putative lateral transfer between fungal species. Until now, there was evidence of horizontally transferred helitrons in insect viruses [5], but to our knowledge this is the first report dealing with the presence of viral domains inside helitron transposons. The presence of these domains in both of the *P. ostreatus* helitron families reinforces their putative role in these transfer events, although reconstructing the phylogenetic history of these elements remains difficult. Based on our data, we hypothesize a putative scenario in which helitrons could have been repeatedly transferred to the fungal kingdom. This horizontal transfer might have been related to previous viral infections of species belonging to the fungal, plant and animal kingdoms with shared ecological niches.

## Conclusion

*P. ostreatus* helitrons display structural and enzymatic features similar to those described in other eukaryotic helitrons. Our results show that *P. ostreatus* helitrons do not capture host genes or gene fragments, as is described in plant and animal helitrons. The occurence of genes probably captured from other hosts inside the helitrons boundaries pose the hypothesis that an ancient horizontal transfer mechanism could have taken place. The viral domains in some of these genes and the polyphyletic origin of RepHel helicases in the eukaryotic kingdom suggests that virus could have played a role in a putatve lateral transfer of helitrons within the eukaryotic kingdom. However, additional data is necessary to support this statement

The high similarity of some elements present in both *Pleurotus* families, along with the transcriptional activity of the RepHel helicases suggests that helitrons are still active in the genome of *P. ostreatus*. The similarities found between *cap* genes as well as their expression profiles suggest that a functional divergence could have occurred, leading to the maintenance or suppression of their activities which could confer a possible advantage for either, the helitron or the host genome.

## Methods

### In silico analysis

#### Structure-based identification of P. ostreatus helitrons

The unmasked assembled genomes of the *P. ostreatus* monokaryotic strains PC15 and PC9 were obtained from the MycoCosm database [28]. The specific web repositories for both genomes are http://genome.jgi-psf.org/PleosPC15_2/PleosPC15_2.home.html, for PC15 and http://genome.jgi-psf.org/PleosPC9_1/PleosPC9_1.home.html for PC9. Both strains were obtained after de-dikaryotization of the strain N001 [47] and are deposited in the Spanish Type Culture collection (PC9. CECT20311 and PC15: CECT20312). The program HelSearch [36] was used to analyze the genomic sequences using the eukaryotic consensus 3′- end helitron structure: a minimum of 6 hairpin pairs (two mismatches allowed) located upstream of a 3′ CTRR motif, a 2-4-bp hairpin loop, and 5–8 bp between the hairpin and the 3′CTRR terminal end. The elements detected by HelSearch were classified and aligned into families according to the conservation of their 3′ ends (30 bp with at least 80% identity). The alignment files produced by HelSearch were manually inspected using MEGA5 [48] to identify the 5′ and 3′ boundaries of each helitron. Elements displaying unclear 3′ boundaries were not used for further analysis. Intact helitrons were defined as elements displaying 5′ and 3′ ends, while truncated elements were defined as those containing an intact 3′ end but not a conserved 5′ end.

#### Homology-based identification of putative autonomous helitrons

The alignment files produced by HelSearch (*.aln files) were processed using Python scripts to obtain the 5′ upstream regions of each helitron end (helend) structure of all the aligned sequences (3600 bp). The genomic sequences were translated to proteins using the three forward reading frames and subjected to a Batch CD Search (plus and minus strands, p < 0.01) [49]. Elements containing Helitron helicase-like (Pfam PF14214) and PIF1-like helicase (Pfam PF05970) domains within the 5′ and 3′ boundaries were considered to be putative autonomous helitrons. Additional *P. ostreatus* helitron-specific helicases were obtained by TBLASTN searches (with a cutoff E-value <10^−5^) using the above mentioned functional domains as queries. Filtered gene models predicted by the JGI and classified as PIF1/DDR3 helicases according to the EuKaryotic Orthologous Groups (KOG) database [50] were also incorporated into the analysis. *P. ostreatus* helitron-specific helicases were aligned using Clustal Omega [51]. The alignments were extended upstream and downstream of the 5′ and 3′ ends to identify the helitron boundaries.

#### Helitron classification

Elements displaying a nucleotide similarity of 80% or higher in the 30-bp 3′ end were considered to be in the same family. Elements that met this requirement but had a similarity of lower than 80% in the 5′ 30-bp end were classified as a subfamily, according to [36]. Helitrons were named using “HELPO” (**Hel**itron ***P****leurotus****o****streatus*) to define the TE class and species, followed by two numbers to define the family and subfamily assignment (i.e., HELPO1.2 belongs to family 1 and subfamily 2). Upright letters are used when referring to families and subfamilies, and italics are used for specific copies (i.e., the HELPO1.1 subfamily vs the *HELPO1.1* element). Putative autonomous elements are shown in uppercase letters, and non-autonomous elements are shown in lowercase letters.

#### Helitron gene capture

Full-length genes present within intact helitrons were analyzed using the JGI browser [28]. Predicted gene models (except RepHel helicases) were considered to be captured genes. The presence of these genes in other fungi was analyzed using BLASTX searches of the MycoCosm and NCBI databases (with a cutoff E-value <10^−10^). In addition, BLASTN searches were performed on *P. ostreatus* PC15 assembled scaffolds to find captured gene fragments (hits that were greater than 50 bp and showed more than 95% identity below a cutoff E-value <10^−5^ were considered to be significant). The promoter regions of the captured genes were examined from the start of the RepHel helicase ORF to the start of the captured gene. These regions were subjected to BLASTN searches against the MycoCosm and ViroBlast [52] databases (cut-off E-value <10 ^-5^).

#### RNA-seq data analysis

The *P. ostreatus* strain N001 was cultured for 8 days on a solid SMY medium (10 g/L saccharose, 10 g/L malt extract, 4 g/L yeast extract, 15 g/L agar) at 24°C in the dark. RNA-seq data from N001 were used to analyze the transcriptional activity of the helitrons and their captured genes. SOLiD RNA-seq reads were mapped to the *P. ostreatus* PC15 (assembled into 11 scaffolds) and PC9 (assembled into 572 scaffolds) genome sequences using TopHat [53], allowing multiple mapping when identical alignment scores where obtained The RPKM method was used to evaluate the transcriptional levels of the helitrons. The IGV tool [33] was used to check for the presence of RNA-seq reads mapping inside the helitron boundaries.

### The search for Helitron-like helicases in fungi and other eukaryotes

A TBLASTN search was carried out against the whole fungal MycoCosm database (unmasked assembly scaffolds with a cutoff E-value <10^−5^) [28] using the two helitron conserved domains (PF14214 and PF05970) as queries. The results were considered to be an indicator of the presence or absence of putative autonomous helitrons in the different fungal species.

Simultaneously, protein models annotated as DNA helicase PIF1/RRM3 (KOG0987) at the Cluster of Orthologous Groups database were downloaded (2175 sequences from 284 fungal genomes) and subjected to a Batch Conserved Domain Database Search using a cut-off E-value <10^−5^. Elements carrying the PF14214 and PF05970 domains were kept for further analysis. The eukaryotic putative autonomous helitrons deposited in Repbase [54] (213 sequences) were downloaded, translated to protein sequences using the three forward reading frames and analyzed as mentioned above. When both searches were combined, the result was a representative group of eukaryotic RepHel helicases that was used for phylogenetic analysis.

### Phylogenetic reconstruction of RepHel helicases

Sequences were aligned using the PhylomeDB pipeline [55]. In brief, three different alignment algorithms were used: MUSCLE v3.8 [56], MAFFT v6.712b [57], and Kalign [58], in the forward and reverse directions (i.e., using the Head or Tail approach) [59]. The six resulting alignments were then combined with M-COFFEE [60] and trimmed with trimAl v1.3 [61] to remove gappy regions and regions that were inconsistent across the reconstructed alignments (with a consistency-score cut-off of 0.1667 and a gap-score cut-off of 0.9). Next, maximum likelihood (ML) trees were reconstructed. First, a tree topology estimated by neighbor joining with BioNJ [62] was used to infer the likelihood of seven different evolutionary models (JTT, LG, WAG, Blosum62, MtREV, VT and Dayhoff). The best model fitting data as determined by the AIC (Akaike’s Information Criterion) [63] were used to derive ML trees using phyML v 3.0 with four rate categories and inferring invariant positions from the data [64]. Branch support was computed using an aLRT (approximate likelihood ratio test) based on a chi-square distribution. The tree figures were produced using ETE v2 [65].

### Experimental analyses

#### Strains and culture conditions

The *P. ostreatus* monokaryotic strains PC15 and PC9 and the dikaryotic strain N001 were grown in triplicate on a submerged SMY medium. Shaking cultures (130 rpm) were kept in the dark at 24°C for 8 days.

#### Nucleic acid extraction and reverse transcription

Total RNA was extracted from ~200 mg of deep frozen tissue using the Fungal RNA E.Z.N.A. Kit (Omega Bio-Tek, Norcross, GA) and treated with 1 U of RQ1 DNase (Promega, Madison, WI) per μg of RNA. The RNA integrity was estimated using denaturing electrophoresis on 1% (w/v) agarose gels. The nucleic acid concentrations were measured with a Qubit 2.0 fluorometer (Life Technologies), and the purity of the total RNA was estimated using the 260/280 nm absorbance ratio on a NanoDrop™ 2000 (Thermo Scientific) machine. The total RNA (225 ng) was reverse-transcribed into cDNA in a 20-μl volume using the iScript cDNA Synthesis kit (Bio-Rad, Alcobendas, Spain).

#### Real-time PCR

The amplifications were performed using a Bio-Rad CFX96 thermal cycler. SYBR green fluorescent dye was used to detect the product amplification. Each reaction was set to a final volume of 20 μl and contained 1X IQ SYBR green Supermix from Bio-Rad, 300 nM forward and reverse primers (Additional file 6: Table S4), and 1 μl of a 1:20 dilution of RT product in nuclease-free water. The amplification program consisted of 5 min at 95°C, 40 cycles of 15 s at 95°C and 30 s at 60°C, followed by 1 min at 95°C, 1 min at 65°C with a final melting curve with increments of 0.5°C every 5 s in a linear gradient of 65 to 95°C. High-temperature fluorescence acquisition (72°C) was performed to eliminate the impact of the PCR artifacts in cDNA quantification, and the absence of these artifacts was confirmed by a melting-curve analysis. A baseline correction and crossing-point (Cp) acquisition were performed using Bio-Rad’s CFXManager. The reactions were performed in triplicate in 96-well microtiter plates. NRTs (non-retrotranscribed controls) and NTCs (no-template controls) were included for each primer set. The amplification efficiencies were sample-estimated by a linear regression from a window-of-linearity set in the exponential phase of the fluorescence history plotted in log scale using the LinReg tool [66]. Raw Cp values were efficiency corrected, and any signal of genomic DNA background was removed using GENEX (http://www.multid.se.). The transcription level of each gene of interest (GOI) was calculated as a relative quantity (equation 1) using *pep* as an internal standard.
1

### Availability of supporting data

The data sets supporting the results of this article are included within the article and its additional files.

## Electronic supplementary material

Additional file 1: Table S1: Summary of helitron-like 3′- terminal ends found by HelSearch. (PDF 86 KB)

Additional file 2: Figure S1: Predicted functional domains of *P. ostreatus* RepHel proteins. The conserved motifs of the Rep catalytic core described by (Jurka, 2007) are shown in black (A). The seven domains of the SF1 helicase superfamily found in helitrons of other species (Feschotte and Pritham, 2006) are shown in B. Black . represent more than 60 % similarity. The phylogenetic tree was constructed using MUSCLE and PhyML. HELITRON1 OS = Oryza sativa helitron. (PDF 32 KB)

Additional file 3: Text S1: Supplementary methods. (DOC 30 KB)

Additional file 4: Table S3: Matrix of nucleotide similarity between intact copies of HELPO1 family captured genes. (PDF 15 KB)

Additional file 5: Dataset S1: Description of conserved domains found in *P. ostreatus* helitrons. (XLS 20 KB)

Additional file 6: Table S4: Primers used for RT-qPCR expression analyses. (PDF 17 KB)

Additional file 7: Figure S2: Phylogenetic reconstruction of eukaryotic Helitron_like helicase domain. Green represents helitrons from the Plant kingdom, yellow from the Animal kingdom and blue from the Fungal kingdom. Light blue represents Class Basidiomycetes and dark blue Class Ascomycetes. (PDF 186 KB)

Additional file 8: Table S2: Similarity between intact copies of the HELPO1 and HELPO2 families in PC15 genome. (PDF 236 KB)
