# Supplementary material for: Highly expressed captured genes and cross-kingdom domains present in Helitrons create novel diversity in Pleurotus ostreatus and other fungi
Source: BMC Genomics. 2014 Dec 5;15(1):1071. doi: 10.1186/1471-2164-15-1071 (PMC4289320; doi:10.1186/1471-2164-15-1071)
Supplement: Supplementary file 1 — Additional file 1: Table S1: Summary of helitron-like 3′- terminal ends found by HelSearch. (PDF 86 KB) [file 12864_2014_6868_MOESM1_ESM.pdf]

Table S1. Summary of helitron-like 3'- terminal ends found by HelSearch

Hairpin family	PC9 repeats	Location PC9 (*)	PC15 repeats	Location PC15 (*)
ACATTCGA_TG_TCGAATGT	2	scaffold_002:1177418-1181018 (+)	2	scaffold_04:2285016-2288616 (+)
		scaffold_002:1191260-1194860 (-)		scaffold_04:2297555-2301098 (-)
AGTGAT_GA_ATCACT	2	scaffold_002:196554-200154 (+)	2	scaffold_04:3240741-3244341 (+)
		scaffold_002:232065-235665 (-)		scaffold_04:3286520-3290120 (-)
CCCGTGC_TTC_GCACGGG	3	scaffold_091:1-659 (+)	2	scaffold_07:1391507-1395107 (+)
		scaffold_006:1348145-1351745 (-)		scaffold_07:1521433-1525033 (-)
		scaffold_004:2670410-2674010 (-)		
CCGTGC_GTA_GCACGG	4	scaffold_142:1-633 (+)	10	scaffold_01:1414547-1418047 (+)
		scaffold_478:273-3873 (-)		scaffold_01:3751228-3754828 (+)
		scaffold_007:1079010-1082610 (+)		scaffold_01:4537480-4541080 (-)
		scaffold_044:2511-6111 (-)		scaffold_02:1934771-1938371 (-)
				scaffold_05:1418338-1421838 (-)
				scaffold_07:378958-382559 (+)
				scaffold_07:2179834-2183434 (+)
				scaffold_08:26455-30055 (-)
				scaffold_08:423543-427143 (-)
				scaffold_11:760623-764223 (+)
TCTTAG_CC_CTAAGA	2	scaffold_006:1916618-1920218 (+)	2	scaffold_07:3019518-3023118(+)
		scaffold_006:1959378-1962978(-)		scaffold_07:3057094-3060694 (-)
AACCAG_GGC_CTGGTT			2	scaffold_06:103948-107548 (-)
				scaffold_06:167219-170819 (+)
AAGTGTG_CA_CACACTT			2	scaffold_06:1808078-1809368 (+)
				scaffold_08:1123002-1126602 (+)
CGTAGCCAC_ACT_GTGGCTACG			4	scaffold_02:2341430-2345030 (-)
				scaffold_02:2382940-2385356 (-)
				scaffold_02:2383133-2385356 (-)
				scaffold_02:2383195-2385356 (-)
CTTGTC_GA_GACAAG			2	scaffold_04: 387277-390877 (+)
				scaffold_04:591329-594929 (-)
GGGCAT_CG_ATGCCC			2	scaffold_06:350706-354306(-)
				scaffold_06:2250480-2254080 (-)
TCAGGG_CTT_CCCTGA			2	scaffold_02:1198298-1201899 (-)
				scaffold_07:41882-45482 (+)
AAATGC_CG_GCATTT	2	scaffold_212:206-3806 (+)		
		scaffold_003:2776155-2779755 (+)		
GGACGG_TAG_CCGTCC	2	scaffold_105:1-3039 (+)		
		scaffold_23:289293-292893 (-)		
TGTGGA_TGAG_TCCACA	2	scaffold_538:1-401 (+)		
		scaffold_007:1677183-1680783 (-)		
TTGCTC_ATC_GAGCAA	2	scaffold_011:11207-14807 (-)		
		scaffold_011:11359-14959 (-)		
**Total number of repetitions**	**21**		**32**	
