# Supplementary material for: Highly expressed captured genes and cross-kingdom domains present in Helitrons create novel diversity in Pleurotus ostreatus and other fungi
Source: BMC Genomics. 2014 Dec 5;15(1):1071. doi: 10.1186/1471-2164-15-1071 (PMC4289320; doi:10.1186/1471-2164-15-1071)
Supplement: Supplementary file 2 — Additional file 2: Figure S1: Predicted functional domains of P. ostreatus RepHel proteins. The conserved motifs of the Rep catalytic core described by (Jurka, 2007) are shown in black (A). The seven domains of the SF1 helicase superfamily found in helitrons of other species (Feschotte and Pritham, 2006) are shown in B. Black . represent more than 60 % similarity. The phylogenetic tree was constructed using MUSCLE and PhyML. HELITRON1 OS = Oryza sativa helitron. (PDF 32 KB) [file 12864_2014_6868_MOESM2_ESM.pdf]

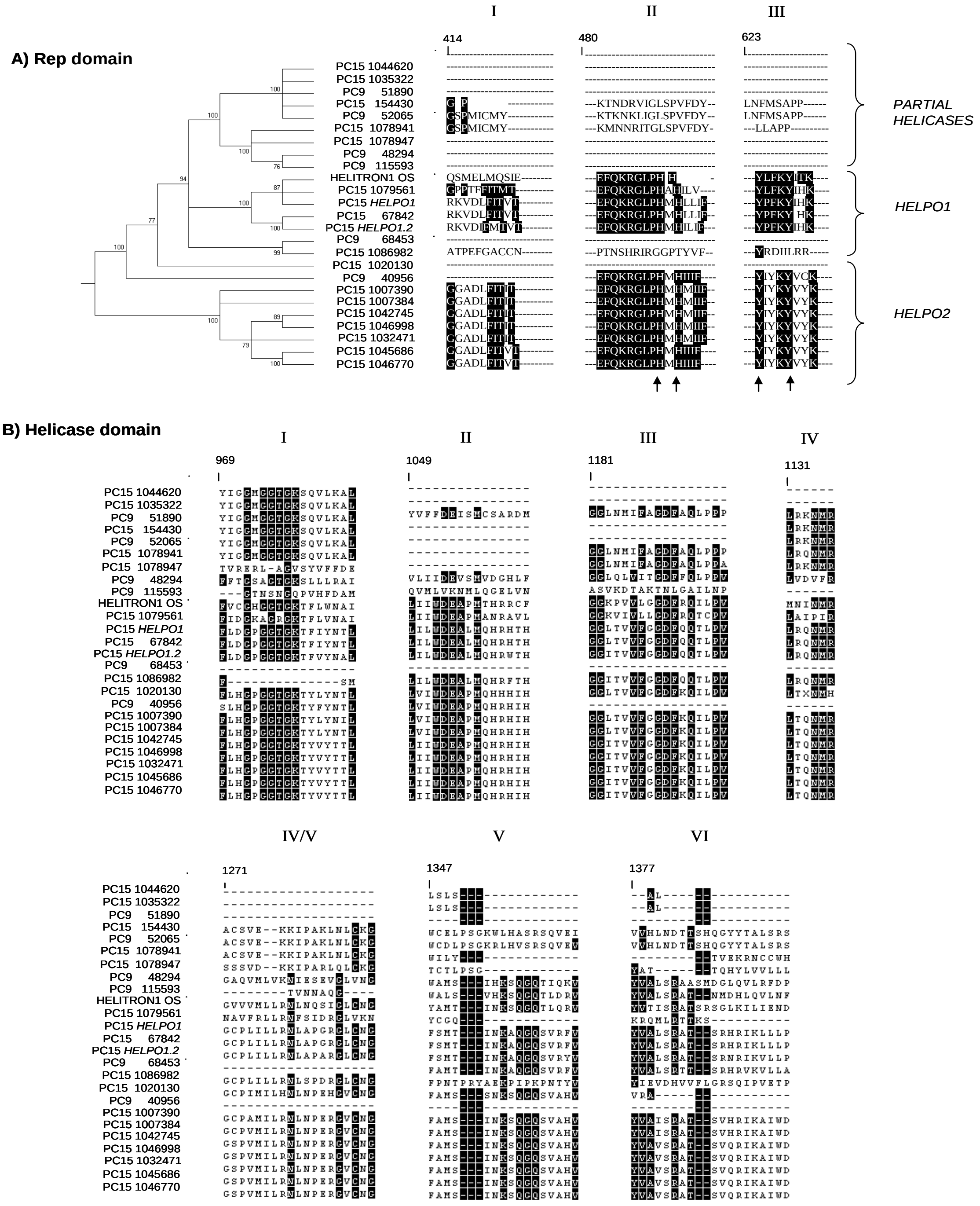

Figure S1. **Predicted functional domains of *P.ostreatus* RepHel proteins**. The conserved motifs of the Rep catalytic core described by (Jurka, 2007) are shown in black (A). The seven domains of the SF1 helicase superfamily found in helitrons of other species (Feschotte and Pritham, 2006) are shown in B. Black represents more than 60 % similarity. The phylogenetic tree was constructed using MUSCLE and PhyML. HELITRON1 OS = *Oryza sativa* helitron.
