# Supplementary material for: Highly expressed captured genes and cross-kingdom domains present in Helitrons create novel diversity in Pleurotus ostreatus and other fungi
Source: BMC Genomics. 2014 Dec 5;15(1):1071. doi: 10.1186/1471-2164-15-1071 (PMC4289320; doi:10.1186/1471-2164-15-1071)
Supplement: Supplementary file 3 — Additional file 3: Text S1: Supplementary methods. (DOC 30 KB) [file 12864_2014_6868_MOESM3_ESM.doc]

**SUPPLEMENTARY METHODS**

**Phylogenetic reconstructions.** Sequence analyses were performed using MEGA5. Alignments were performed with Clustal Omega. Phylogenetic analysis was performed on JGI filtered-protein sequences using the Phylogeny.fr platform [1] and comprised the following steps: a multiple sequence alignment was generated using MUSCLE v3.7 [2]. After alignment, ambiguous regions were removed with Gblocks v0.91b [3] using the default settings. Phylogenetic tree was reconstructed using the maximum likelihood method implemented in the PhyML program v3.0 aLRT [4, 5]. Graphical representation and edition of the phylogenetic tree were performed with TreeDyn v198.3 [6].

**Whole genome alignment**. A whole genome alignment between PC15 and PC9 genomes was performed using the Mercator and MAVID pipeline [7]. The adjacent regions of each PC15 helitron shown in Table 1 were analyzed (50 kb upstream and downstream). The locations of every gene placed in these 50kb windows were used to extract individual alignments between PC15 and PC9. The alignments were parsed using Python scripts, and a break of gene colinearity was considered when a gene was present in PC15 but absent in PC9. A gene was considered absent when the alignment length of PC9 was lower than 20% of the length in the PC15 locus, and the frequency of colinearity breaks per every 50kb window was calculated. The same procedure was applied to the full chromosome I, and results were used as a reference estimation of the whole genome. This chromosome was chosen because it is almost fully assembled into a single scaffold in both genomes. The PC15 loci absent in PC9 were used as BlastN query (cutoff E-value < 10-15 and 95% similarity) to check if they were present at a different location. In addition, the same loci were used in a BlastX search (cutoff E-value < 10-5) in the Repbase peptide database [8] to check if they matched to other transposable elements.

**REFERENCES**

1. Dereeper A, Guignon V, Blanc G, Audic S, Buffet S, Chevenet F, Dufayard JF, Guindon S, Lefort V, Lescot M *et al*: **Phylogeny.fr: robust phylogenetic analysis for the non-specialist**. *Nucleic Acids Res* 2008, **36**:W465-W469.

2. Edgar RC: **MUSCLE: multiple sequence alignment with high accuracy and high throughput**. *Nucleic Acids Res* 2004, **32**(5):1792-1797.

3. Castresana J: **Selection of conserved blocks from multiple alignments for their use in phylogenetic analysis**. *Mol Biol Evol* 2000, **17**(4):540-552.

4. Anisimova M, Gascuel O: **Approximate likelihood-ratio test for branches: A fast, accurate, and powerful alternative**. *Syst Biol* 2006, **55**(4):539-552.

5. Guindon S, Dufayard JF, Lefort V, Anisimova M, Hordijk W, Gascuel O: **New Algorithms and Methods to Estimate Maximum-Likelihood Phylogenies: Assessing the Performance of PhyML 3.0**. *Syst Biol* 2010, **59**(3):307-321.

6. Chevenet F, Brun C, Banuls AL, Jacq B, Christen R: **TreeDyn: towards dynamic graphics and annotations for analyses of trees**. *Bmc Bioinformatics* 2006, **7**.

7. Dewey CN: **Aligning multiple whole genomes with Mercator and MAVID**. *Methods in molecular biology (Clifton, NJ* 2007, **395**:221-236.

8. Jurka J: **Repbase Update - a database and an electronic journal of repetitive elements**. *TIG* 2000, **16**(9):418-420.
