# Supplementary material for: Highly expressed captured genes and cross-kingdom domains present in Helitrons create novel diversity in Pleurotus ostreatus and other fungi
Source: BMC Genomics. 2014 Dec 5;15(1):1071. doi: 10.1186/1471-2164-15-1071 (PMC4289320; doi:10.1186/1471-2164-15-1071)
Supplement: Supplementary file 4 — Additional file 4: Table S3: Matrix of nucleotide similarity between intact copies of HELPO1 family captured genes. (PDF 15 KB) [file 12864_2014_6868_MOESM4_ESM.pdf]

Table S3. Matrix of nucleotide similarity between intact copies of HELPO1 family captured genes.

	capA	***capA***	***capD***	***capC***	***capE***	***capB***	***capB***	***capF***	***capA2***
capA	100.00	99.53	44.80	53.60	54.57	43.49	43.49	38.07	45.28
capA	99.53	100.00	44.83	53.36	54.72	43.43	43.43	37.91	45.33
capD	44.80	44.83	100.00	74.91	54.01	40.12	40.02	30.80	41.02
capC	53.60	53.36	74.91	100.00	62.16	46.08	46.37	41.71	49.33
capE	54.57	54.72	54.01	62.16	100.00	42.35	42.18	41.88	46.48
capB	43.49	43.43	40.12	46.08	42.35	100.00	99.23	47.54	60.84
capB	43.49	43.43	40.02	46.37	42.18	99.23	100.00	47.54	60.73
capF	38.07	37.91	30.80	41.71	41.88	47.54	47.54	100.00	74.98
capA2	45.28	45.33	41.02	49.33	46.48	60.84	60.73	74.98	100.00
