# Supplementary material for: Highly expressed captured genes and cross-kingdom domains present in Helitrons create novel diversity in Pleurotus ostreatus and other fungi
Source: BMC Genomics. 2014 Dec 5;15(1):1071. doi: 10.1186/1471-2164-15-1071 (PMC4289320; doi:10.1186/1471-2164-15-1071)
Supplement: Supplementary file 6 — Additional file 6: Table S4: Primers used for RT-qPCR expression analyses. (PDF 17 KB) [file 12864_2014_6868_MOESM6_ESM.pdf]

Table S4. Primers used for RT-qPCR expression analyses.

Name	Sequence	Efficiency	Specificity *
*HELPO1.1*rep.fw	CCAGATGCCGAGATCAAGCTTCG	1.87	*HELPO1* helicases (4)
HELPO1.1 rep.RV	GGCATAATCATGGCAACCTC		
HELPO1.2rep.fw	CCACCAACTCCCACAGAAT	1.86	*HELPO1.2* helicases (2)
HELPO1.2rep.fw	TCTAGCACCCCCGATTTATG		
*HELPO2*_dg.fw	AAACTGCGGACTCCTGAAGA	1.87	*HELPO2* helicases (6)
*HELPO2*_dg.rv	CAGCTGTGGTGCTTCCAGTA		
capA.fw	AAATGGACCCCTCCGTTTAC	1.98	*capA* genes (3) - HELPO1
capA.rv	TTTCTGCAAGGGACCCATAG		
capA2.fw	CCTGTTGTTGCATGATCCAG	1.89	*capA2* gene (1) - HELPO1
capA2.rv	GATGTGCGCCTCAGTAGACA		
capB.fw	GGGCTTGCTGTATTGGAAAA	1.91	*capB* genes (2) - HELPO1.2
capB.rv	TGGGGAGCGAGATAGAATTG		
capC.fw	CACGAGCAATTTTTGCAATG	1.87	*capC* genes (6) - HELPO1.3
capC.rv	GTAAGGGTCCTGAGCAGCAG		
capD.fw	GCAGAGCAGCGAGAGTTTCT	/	*capD* gene (1) - HELPO1.3
capD.rv	AAAATCCCGGTACGTGTTCA		
capF.fw	CATTGGACTGGGAATCTGCT	/	*capF* gene (1) - HELPO1.3
capF.rv	CCCTGCTTTTTGACTTCAGC		
*pep*_fw	CTATCTCGGGAACGGTATATCA	1.89	PC15 V2.0 ID:1092697
*pep*_rv	CCGCTGGTACTGGTACTATAA		

* In parenthesis is shown the number of gene copies in PC15 genome
