# Supplementary material for: Highly expressed captured genes and cross-kingdom domains present in Helitrons create novel diversity in Pleurotus ostreatus and other fungi
Source: BMC Genomics. 2014 Dec 5;15(1):1071. doi: 10.1186/1471-2164-15-1071 (PMC4289320; doi:10.1186/1471-2164-15-1071)
Supplement: Supplementary file 7 — Additional file 7: Figure S2: Phylogenetic reconstruction of eukaryotic Helitron_like helicase domain. Green represents helitrons from the Plant kingdom, yellow from the Animal kingdom and blue from the Fungal kingdom. Light blue represents Class Basidiomycetes and dark blue Class Ascomycetes. (PDF 186 KB) [file 12864_2014_6868_MOESM7_ESM.pdf]

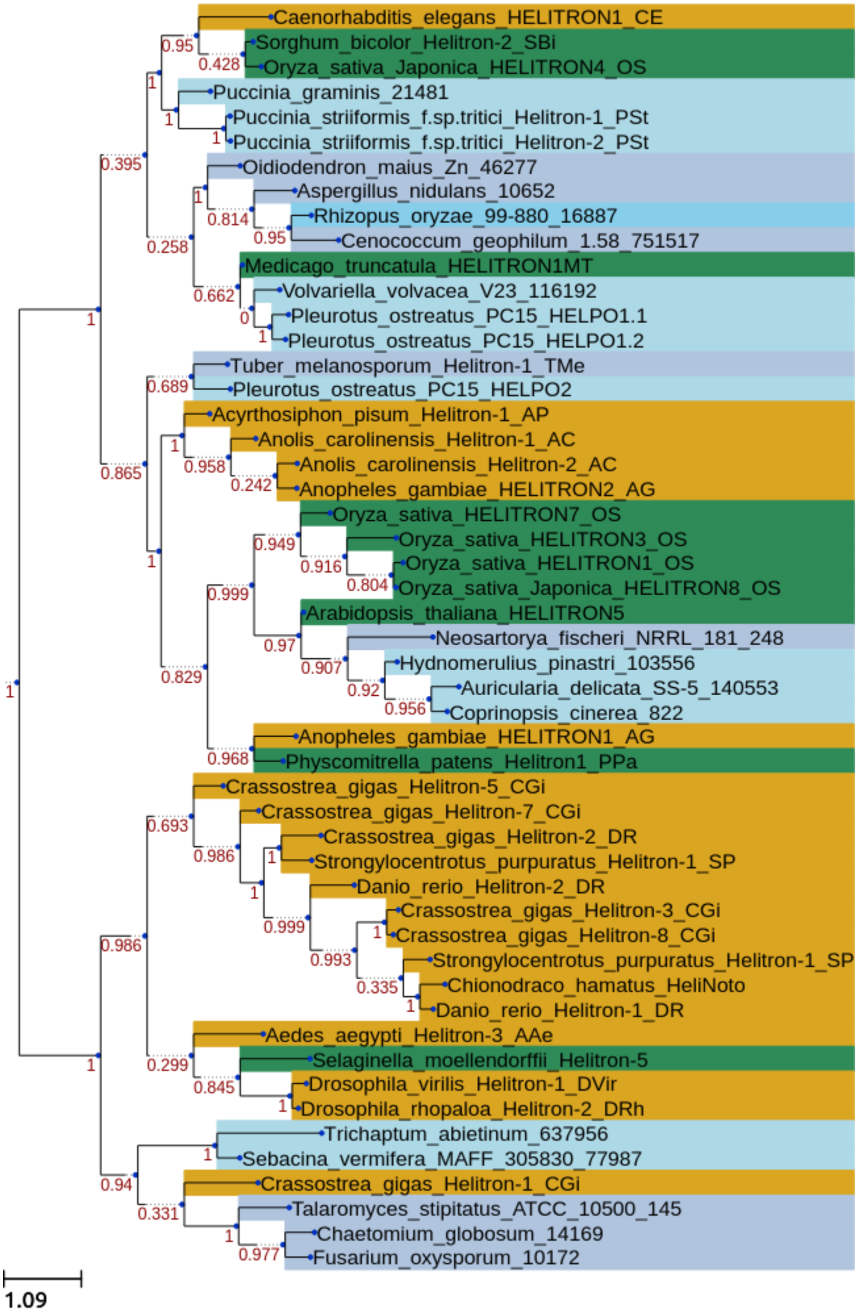

Figure S2. **Phylogenetic reconstruction of eukaryotic Helitron_like helicase domain**. Green represents helitrons from the Plant kingdom, yellow from the Animal s kingdom , and blue from the Fungal kingdom. Light blue represents Class Basidiomycetes and dark blue Class Ascomycetes.
