# Supplementary material for: Highly expressed captured genes and cross-kingdom domains present in Helitrons create novel diversity in Pleurotus ostreatus and other fungi
Source: BMC Genomics. 2014 Dec 5;15(1):1071. doi: 10.1186/1471-2164-15-1071 (PMC4289320; doi:10.1186/1471-2164-15-1071)
Supplement: Supplementary file 8 — Additional file 8: Table S2: Similarity between intact copies of the HELPO1 and HELPO2 families in PC15 genome. (PDF 236 KB) [file 12864_2014_6868_MOESM8_ESM.pdf]

Table S2. Similarity between intact copies of the HELPO1 and HELPO2 families in PC15 genome.

| | | | | | | | | | | | | | | | | |
|---|---|---|---|---|---|---|---|---|---|---|---|---|---|---|---|---|
| helpo1.3_scaffold_08_RC | 100.00 | 96.58 | 96.27 | 96.01 | 96.63 | 96.81 | 96.67 | 47.45 | 47.56 | 47.56 | 47.56 | 47.56 | 40.72 | 40.65 | 37.84 | 37.85 |
| helpo1.3_scaffold_07_2 | 96.58 | 100.00 | 98.63 | 98.50 | 99.06 | 99.02 | 98.89 | 46.94 | 47.05 | 47.05 | 47.05 | 47.05 | 41.21 | 41.15 | 39.33 | 38.42 |
| helpo1.3_scaffold_02_RC | 96.27 | 98.63 | 100.00 | 98.19 | 98.59 | 98.83 | 98.70 | 47.11 | 47.22 | 47.22 | 47.22 | 47.22 | 40.55 | 40.49 | 39.24 | 38.34 |
| helpo1.3_scaffold_01_RC | 96.01 | 98.50 | 98.19 | 100.00 | 98.46 | 98.58 | 98.45 | 47.01 | 47.11 | 47.11 | 47.11 | 47.11 | 40.94 | 40.88 | 39.15 | 38.59 |
| helpo1.3_scaffold_08_42_RC | 96.63 | 99.06 | 98.59 | 98.46 | 100.00 | 98.93 | 98.86 | 47.23 | 47.33 | 47.33 | 47.33 | 47.33 | 40.74 | 40.67 | 39.00 | 38.51 |
| helpo1.3_scaffold_07_1 | 96.81 | 99.02 | 98.83 | 98.58 | 98.93 | 100.00 | 99.87 | 47.23 | 47.33 | 47.33 | 47.33 | 47.33 | 40.87 | 40.81 | 39.32 | 38.51 |
| helpo1.3_scaffold_09_RC | 96.67 | 98.89 | 98.70 | 98.45 | 98.86 | 99.87 | 100.00 | 47.23 | 47.33 | 47.33 | 47.33 | 47.33 | 40.87 | 40.81 | 39.24 | 38.51 |
| HELPO2_scaffold_01 | 47.45 | 46.94 | 47.11 | 47.01 | 47.23 | 47.23 | 47.23 | 100.00 | 100.00 | 100.00 | 100.00 | 100.00 | 50.20 | 50.25 | 50.18 | 49.01 |
| HELPO2_scaffold_06_RC | 47.56 | 47.05 | 47.22 | 47.11 | 47.33 | 47.33 | 47.33 | 100.00 | 100.00 | 100.00 | 100.00 | 100.00 | 50.19 | 50.25 | 50.19 | 49.00 |
| HELPO2_scaffold_07_RC | 47.56 | 47.05 | 47.22 | 47.11 | 47.33 | 47.33 | 47.33 | 100.00 | 100.00 | 100.00 | 100.00 | 100.00 | 50.19 | 50.25 | 50.19 | 49.00 |
| HELPO2_scaffold_08 | 47.56 | 47.05 | 47.22 | 47.11 | 47.33 | 47.33 | 47.33 | 100.00 | 100.00 | 100.00 | 100.00 | 100.00 | 50.19 | 50.25 | 50.19 | 49.00 |
| HELPO2_scaffold_08_2_RC | 47.56 | 47.05 | 47.22 | 47.11 | 47.33 | 47.33 | 47.33 | 100.00 | 100.00 | 100.00 | 100.00 | 100.00 | 50.19 | 50.25 | 50.19 | 49.00 |
| HELPO1.1_scaffold_07_1 | 40.72 | 41.21 | 40.55 | 40.94 | 40.74 | 40.87 | 40.87 | 50.20 | 50.19 | 50.19 | 50.19 | 50.19 | 100.00 | 99.74 | 56.93 | 56.69 |
| HELPO1.1_scaffold_07_2 | 40.65 | 41.15 | 40.49 | 40.88 | 40.67 | 40.81 | 40.81 | 50.25 | 50.25 | 50.25 | 50.25 | 50.25 | 99.74 | 100.00 | 56.91 | 56.71 |
| HELPO1.1_scaffold_05_RC | 37.84 | 39.33 | 39.24 | 39.15 | 39.00 | 39.32 | 39.24 | 50.18 | 50.19 | 50.19 | 50.19 | 50.19 | 56.93 | 56.91 | 100.00 | 60.05 |
| HELPO1.2_scaffold_11 | 37.85 | 38.42 | 38.34 | 38.59 | 38.51 | 38.51 | 38.51 | 49.01 | 49.00 | 49.00 | 49.00 | 49.00 | 56.69 | 56.71 | 60.05 | 100.00 |
